# Manipulation of lignin metabolism by plant densities and its relationship with lodging resistance in wheat

**DOI:** 10.1038/srep41805

**Published:** 2017-02-02

**Authors:** Mengjing Zheng, Jin Chen, Yuhua Shi, Yanxia Li, Yanping Yin, Dongqing Yang, Yongli Luo, Dangwei Pang, Xu Xu, Wenqian Li, Jun Ni, Yuanyuan Wang, Zhenlin Wang, Yong Li

**Affiliations:** 1 State Key Laboratory of Crop Biology, Ministry of Science and Technology, Shandong Agricultural University, Tai’an 271018, Shandong, P. R. China; 2 Agricultural Bureau of Yanzhou District, Jining, Shandong, P. R. China

## Abstract

Increasing plant density is one of the most efficient ways of increasing wheat (*Triticum aestivum* L.) grain production. However, overly dense plant populations have an increased risk of lodging. We examined lignin deposition during wheat stem development and the regulatory effects of plant density using the wheat cultivars shannong23 and weimai8. Plants were cultivated at densities of 75, 225 and 375 plants per m^2^ during two growing seasons. Our results showed that decreasing plant density enhanced culm quality, as revealed by increased culm diameter, wall thickness and dry weight per unit length, and improved the structure of sclerenchyma and vascular bundles by increasing lignification. In addition, more lignins were deposited in the secondary cell walls, resulting in strong lodging resistance. The guaiacyl unit was the major component of lignin and there was a higher content of the syringyl unit than that of the hydroxybenzyl unit. Furthermore, we hypothesised that the syringyl unit may correlate with stem stiffness. We describe here, to the best of our knowledge, the systematic study of the mechanism involved in the regulation of stem breaking strength by plant density, particularly the effect of plant density on lignin biosynthesis and its relationship with lodging resistance in wheat.

Stem lodging in wheat (*Triticum aestivum* L.) is a major agronomic problem that has far-reaching economic consequences^1^,^2^. The Green Revolution has effectively increased lodging resistance and the harvest index through the use of semi-dwarf trait^3^. However, improving the lodging resistance of crops by reducing plant height is only possible up to a certain limit in current crop production. Therefore, determining how to improve stem quality, especially the mechanical strength of the basal culm, has become the main target for increasing crop lodging resistance and thus grain yield.

The plant cell wall provides mechanical support to cells, tissues and the entire plant body^4^. The composition and structure of plant cell walls are ideally suited to the functions they perform. For example, parenchyma cells, which possess primary walls, provide the main structural support in the growing regions of the plant body. Sclerenchyma cells, which have both primary and thick secondary walls, provide the major mechanical support in the mature regions of the plant body^5^. During secondary cell wall synthesis, lignins are deposited in the carbohydrate matrix of the cell wall, providing the plant body with strong mechanical support to enable the plant to grow upwards^6^. Lignin content and composition are important factors that affect the cell wall stiffness and the mechanical strength of the plant body^7^. The ability to synthesize lignin has been essential in the evolutionary adaptation of plants from an aquatic environment to land^8^,^9^. Lignins are irreversible endpoints of a major metabolic pathway in the phenylpropane pathway. Previous studies have demonstrated that lignins are important to the mechanical strength of in other plant species. For example, Sherry *et al*. conducted a quantitative trait locus (QTL) analysis of stalk strength in four Maize populations. They showed that candidate genes with overlapping QTL confidence intervals included those involved in lignin synthesis^10^. In addition, in brown midrib mutants of maize, lignin content was extensively reduced with decreasing the stalk mechanical strength^11^. Significant correlations were similarly found between lignin accumulation and the mechanical strength of wheat (*Triticum aestivum* L.) and common buckwheat (*Fagopyrum esculentum* M.)^12^,^13^,^14^.

Lignin deposition in the plant cell walls is not only developmentally regulated but can also be affected by environmental conditions such as biotic and abiotic stresses^15^. During the early stages of lignification, H and G units and a few S units are incorporated into the polymer. Subsequently, coniferyl alcohol and sinapyl alcohol are incorporated during secondary wall formation to form a fusion of G and S units^16^. Plants are frequently exposed to different stresses that may change their lignin content and composition. For example, light itself has an effect on lignin biosynthesis^17^. More photosynthetic products, e.g. glucose, enter the shikimic acid pathway and generate more lignin under sufficient light conditions. Another study also showed that carbon availability related to starch turnover might determine the capacity to synthesise lignin in comparisons of circadian changes in the transcript abundance of lignin biosynthetic genes between wild-type plants and the *sex1* mutant, which exhibits impaired in starch turnover^18^. Lignin composition was also altered in the *sex1* mutants, with an increased proportion of syringyl as compared with guaiacyl and *p-*hydroxyphenyl^18^. Mechanical ingures induce specific reactions in the plant. Electron microscopy analysis has demonstrated that mechanical injures induce the thickening of the xylem the stem cell wall of poplar^19^.These findings indicate that the regulation of lignin accumulation in plants is complex and involves various elements, therefore, the molecular mechanisms that determine the mechanical strength are similarly complex.

Mechanical strength is controlled by quantitative trait loci (QTL)^20^,^21^,^22^, and regulated by growth regulators^23^,^24^, fertiliser^25^, and plant density^26^. Many measures have mainly focused on the morphological characteristics of the crop, including plant height, internode length, and culm diameter. Functional analyses of lignin biosynthetic enzymes, including phenylalanine ammonia-lyase (PAL)^27^, coumarate 3-hydroxylase (C3H)^28^, 4-coumarate: CoA ligase (4CL)^29^, ferulic acid/coniferaldehyde/coniferyl alcohol 5-hydroxylase (F5H)^30^, caffeic acid-3-*o*-methytransferase (COMT)^31^, cinnamoyl CoA reductase (CCR)^32^, and cinnamyl alcohol dehydrogenase (CAD)^33^ have been performed in several plants, inculding the model-plants Arabidopsis and tobacco. However, determining the expression pattern of the genes involved in lignin biosynthesis and lignin monomer ratio in winter wheat, and how winter wheat the responds the cultivation conditions remain to be elucidated.

Increasing plant density is the main ways of improving the grain yield of winter wheat. However, overly dense populations may lead to weak stem characteristics and a consequent lodging with strong wind and rain. Studies of the crop lodging response to plant density have reported inconsistent results. Most studies have demonstrated that lodging is more severe at higher plant densities. For example, one study reported that increasing of the plant density caused significant decreases in the stalk crushing strength, dry weight per unit length, and the diameter of the internode decreased significantly^34^. Other studies also showed that as the plant density increased, the length of the basal internode increased; the mechanical tissue thickeness decreased, the mechanical cell layers and cortical thickness reduced; and the lodging rate increased^35^,^36^. While a study of reepseed demonstrated that lodging resistance was elevated with increased plant density^37^. The response of trees, e.g. poplar, to plant density in terms of diameter and cell wall thickness has been previously reported^38^,^39^. Elucidating how plant density regulates lignin biosynthesis in the basal culm, and its relationship with lodging resistance in wheat is of great importance. Furthermore, the investigation of lignin biosynthesis to improve wheat cultivation techniques is an issue worthy to research. In this study, we performed experiment on two extensively cultivated wheat varieties to investigate the effect of three different planting densities on lignin biosynthesis. We reported an in-depth characterisation of the wheat stem, especially regarding lignin biosynthesis and its spatial expression patterns at different plant population densities. We aimed to clarify the regulatory mechanism of plant densities on lodging resistance in wheat from the aspect of lignin biosynthesis. To the best of our knowledge, we provide the first in-depth characterisation of the regulatory role of plant densities in lignin biosynthesis in wheat. A greater understanding of these issues should provide a theoretical basis to enhance the physical strength of the basal part of the culm internode, with the aim of obtaining a higher grain yield.

## Results

### Effects of plant densities on breaking strength

In both cultivars, from anthesis to the hard dough stage, the breaking strength gradually decreased. Plant density significantly (P < 0.01) influenced the breaking strength of the second basal internodes, which gradually increased with decreasing densities. Compared with the D3 treatment, the D2 treatment significantly increased the breaking strength of the second basal internode by 46.41%, 54.39% and 112.5% from 2012 to 2013 and by 29.97%, 81.64% and 88.14% from 2013 to 2014 (means of the two cultivars) at the anthesis, milk and hard dough stages, respectively. Similarly, the D1 treatment significantly increased the breaking strength by 85.02%, 90.43% and 178.24% and by 65.08%, 163.18% and 178.53% (means of two cultivars) for the respective parameters. The characteristics of the stem second basal internodes of the WM8 plants were increased compared with the SN23 plants in all treatments in both growing seasons (Table 1).

### Morphological characteristics of the basal second internode

To evaluate the differences in stem mechanical strength that respond to plant density, the morphological characteristics of the second basal internode were analysed. The second basal internode characteristics were strongly (P < 0.01) affected by plant density (Table 2). Compared with the D3 treatment, the diameter of the second basal internode of the D1 and D2 plants was approximately 15.46% and 9.9% (mean of two cultivars) larger, respectively, from 2012 to 2013 and approximately 13.31% and 5.57% larger, respectively, from 2013 to 2014. Compared with the D3 treatment, D1 and D2 plants exhibited significant increases in wall thickness of 37.76% and 23.78%, respectively, from 2012 to 2013, and 27.11% and 13.25%, respectively, from 2013 to 2014. Plant density also strongly affected the dry weight per unit length of the second basal internode and increased with reduced plant densities (Table 2). Compared with the D3 treatment, the D2 treatment resulted in an increase in the dry weight per unit length of 38.29% and 36.54%, whereas the D1 treatment resulted in an increase in the dry weight per unit length of 91.75% and 97.71% from 2012 to 2013 and from 2013 to 2014, respectively. The culm characteristics of the two cultivars showed similar trends. The morphological features of the WM8 plants were significantly (P < 0.05) increased compared with the SN23 plants.

### Microscopic structural characteristics of the second basal internode

**Figure 1:**
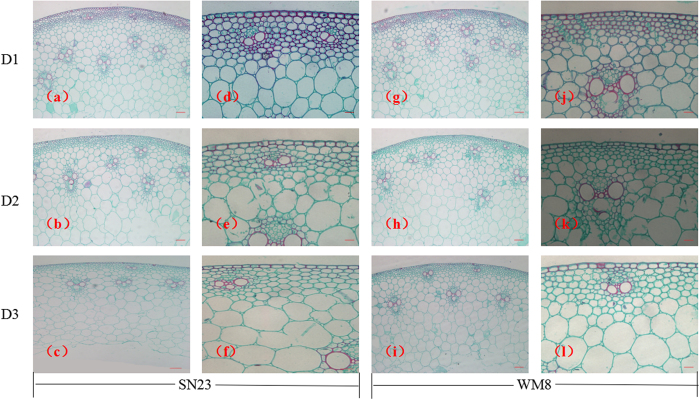
Effects of plant densities on the degree of lignification of the second basal internode of two wheat cultivars. Cross-sections of the second basal internodes of two wheat cultivars stained for lignification with safranin O-fast green. The first two columns indicate SN23; the last two columns indicate WM8; first line, D1 treatment (75 plants m^−2^); second line, D2 treatment (225 plants m^−2^); third line, D3 treatment (375 plants m^−2^). Scale bars: first and third columns, 200 μm; second and fourth columns, 20 μm.

To further identify the factors responsible for the effects of plant densities on the breaking strength, the anatomical structure of the plants was analysed for each of the treatments. In the transverse sections, lignin was observed mainly in the epidermis, sclerenchyma and vascular bundles (Fig. 1). Stain intensity did not differ between the cultivars that received the same treatment. Nevertheless, a greater number of vascular bundles were observed in the WM8 transverse sections than in the SN23 plants in the same visual field. The sclerenchyma of the WM8 plants was also arranged more compactly compared with the SN23 plants. The wheat second basal internodes showed significant differences in their degree of lignification between the treatments. The lignification area in the D1 plants was remarkably larger than that seen in the D2 and D3 plants, and a greater number of vascular bundles were observed in the D1 and D2 plants compared with the D3 plants (Fig. 1).

**Figure 2:**
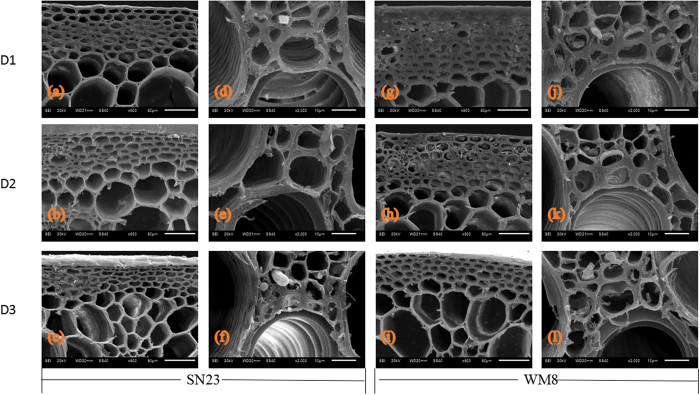
Scanning electron microscopy of cross-sections of the second basal internode of plants grown at different plant densities. Transverse sections of the second basal internode were obtained at 10 days after anthesis. The first two columns indicate SN23; the last two columns indicate WM8; first line, D1 treatment (75 plant m^−2^); second line, D2 treatment (225 plants m^−2^); third line, D3 treatment (375 plants m^−2^). The first and third columns reveal details of the sclerenchyma; the second and fourth columns show details of the vascular bundle. Scale bars: first and third columns, 50 μm; second and fourth columns, 10 μm.

Furthermore, to determine any anatomical changes among the plant density treatments, we analysed basal second internode cross-sections by scanning electron microscopy. Plant densities strongly affected the sclerenchyma and the structure of vascular bundles (Fig. 2). As plant density decreased, the abundance of sclerenchyma cells in the cortex increased, the walls were thicker and the arrangement was more compact. Plant density had a similar effect on the vascular bundles. Compared with the D3 treatment, the vascular bundles of the plants that received the D1 treatment were stronger structures with thicker cell walls and a more compact arrangement of sclerenchyma cells. The structure of the sclerenchyma and vascular bundles in the WM8 plants was slightly superior compared with the SN23 plants.

### Effects of plant densities on lignin accumulation

**Figure 3:**
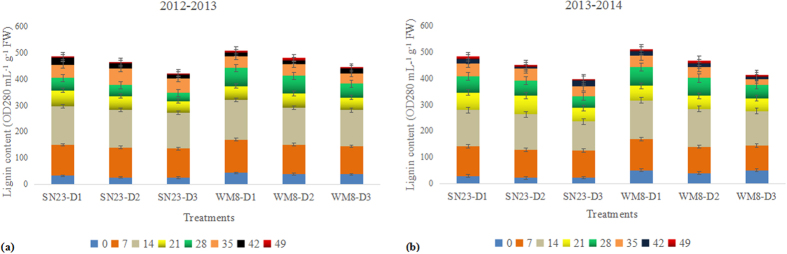
Effect of plant densities on lignin accumulation in the second basal internode of two wheat cultivars. Different colours represent the days after the second internode formation in the 2012–2013 (**a**) and 2013–2014 (**b**) growing seasons. FW, fresh weight.

Based on the different effects that the different plant densities had on the degree of lignification, lignin accumulation during wheat stem development was determined. A significant difference in lignin concentration was observed between treatments; lignin accumulation increased with decreasing plant density. The total lignin accumulation of the SN23 plants that received the D1 or D2 treatment was increased by 18.69% and 12.26% (means of two years), respectively, compared with the D3 plants, whereas these values were 18.74% and 10.24%, respectively, in WM8 plants. Furthermore, lignin accumulation during wheat stem development was inconstant (Fig. 3). A relatively faster accumulation rate was noted 0 to 35 days after the formation of the second internode (jointing to early grain filling) compared with 42 to 49 days (later grain filling to hard dough stage). The data also showed that the second basal internode of WM8 plants accumulated more lignin than that of SN23 plants in both growing seasons.

### Effects of plant densities on lignin monomers

We successfully separated the three lignin monomers using UPLC-MS/MS (Supplementary Fig. S2). In both genotypes, guaiacyl units formed the main structure, followed by syringyl and finally hydrobenzyl units. Plant density had significant effects on both total lignin and monomer contents.

**Figure 4:**
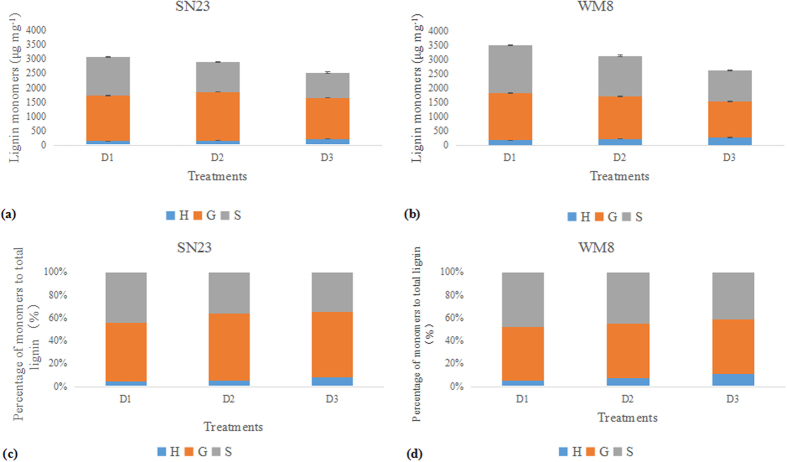
Lignin monomer content and percentage. The lignin building units hydroxyphenyl (blue), guaiacyl (yellow) and sinapyl (grey) were determined in mature wheat by the alkaline nitrobenzene oxidation of purified cell walls. Data of stacked columns are presented as the means ± the SE of three replicates.

The total lignin monomer contents of the WM8 plants were higher than those of the SN23 plants. In addition, the plants that received the D3 treatment showed the lowest total released monomers. Compared with the D3 treatment, the total monomer contents of the SN23 plants that received the D1 and D2 treatments were 17% and 10% higher, respectively, and the total monomer contents of the WM8 plants were 13% and 8% higher, respectively (Fig. 4a,b). Both cultivars showed a similar variation in the monomers that were released depending on the planting density. Increasing plant density led to the increased production of H monomers at the expense of S monomers, and the G monomer varied slightly (Fig. 4c,d).

### Effects of plant densities on the expression pattern of genes involved in lignin biosynthesis

**Figure 5:**
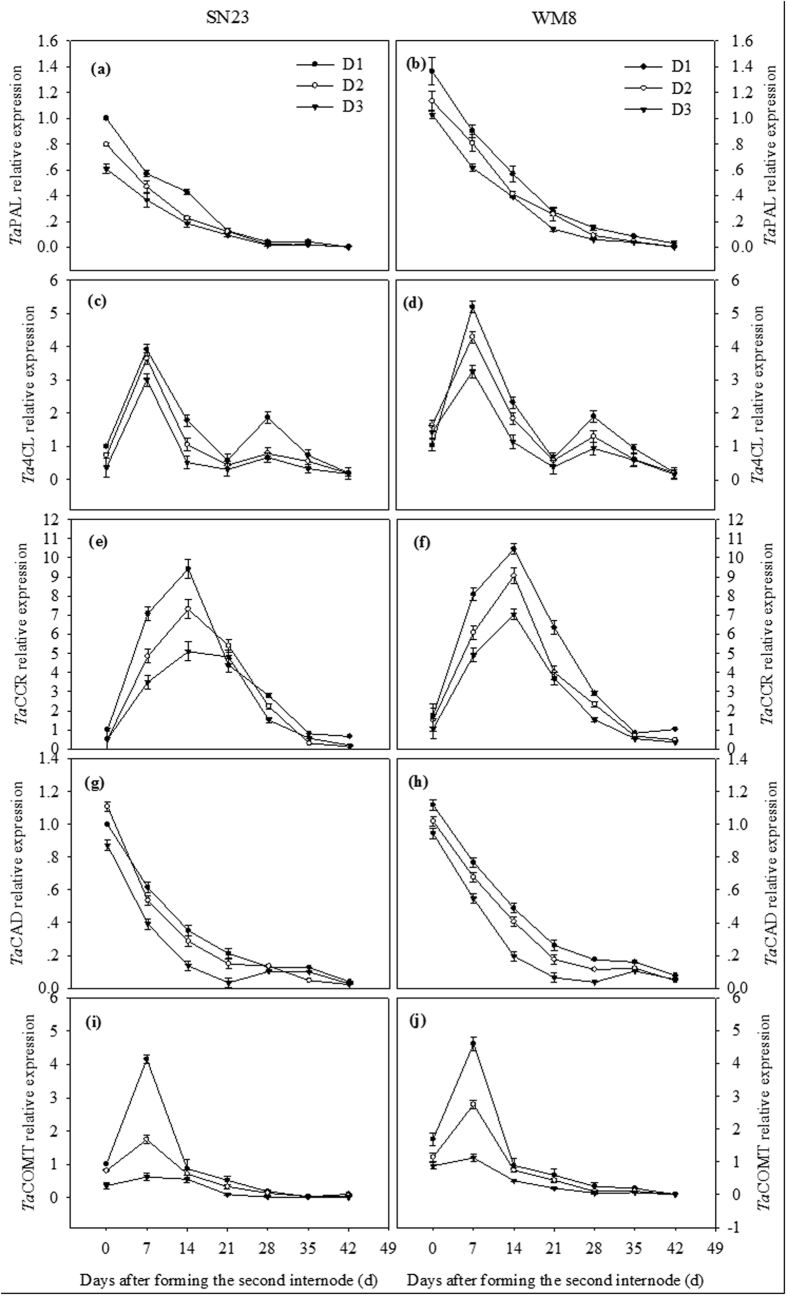
The expression profile of lignin biosynthetic genes analysed by quantitative reverse transcription-PCR.

To illuminate the genes that were likely to be involved in wheat stem lignification, we compared the transcript abundance of the phenylpropanoid gene during wheat stem development by quantitative PCR (Fig. 5). Several distinct spatial expression profiles were observed among the analysed wheat genes. As the stem developed, the relative expression of the *Ta*PAL gene gradually decreased. *Ta*4CL gene expression reached its peak 14 days after the second internode formation followed by a second peak at 28 days. The relative expression of the *Ta*CCR gene was highest at 0 to 14 days and then gradually decreased. The relative expression of the *Ta*CAD gene decreased to its minimum at 21 days after the formation of the second internode, then slightly increased at 28 days, and decreased again at 42 days. The relative expression of the *Ta*COMT gene was highest at 0 to 14 days after the formation of the second internode, decreased sharply and was minimally detectable at the late growth stage. The highest expression levels in the second basal internode samples were concomitant with the increase in lignin content during stem development. Plant density has strong regulating effects on the expression of lignin biosynthesis genes. Such genes were expressed at relatively higher levels in the D1 treatment, which might explain the higher levels of lignin monomers in the D1 treatment. Overall, the gene expression levels of the WM8 plants were higher than those of the SN23 plants at all stem developmental stages. The lower expression levels of lignin biosynthesis genes approximately at maturity could be due to their preferential expression in young tissues.

### Correlation analysis between breaking strength and morphological characteristics as well as lignin content

**Figure 6:**
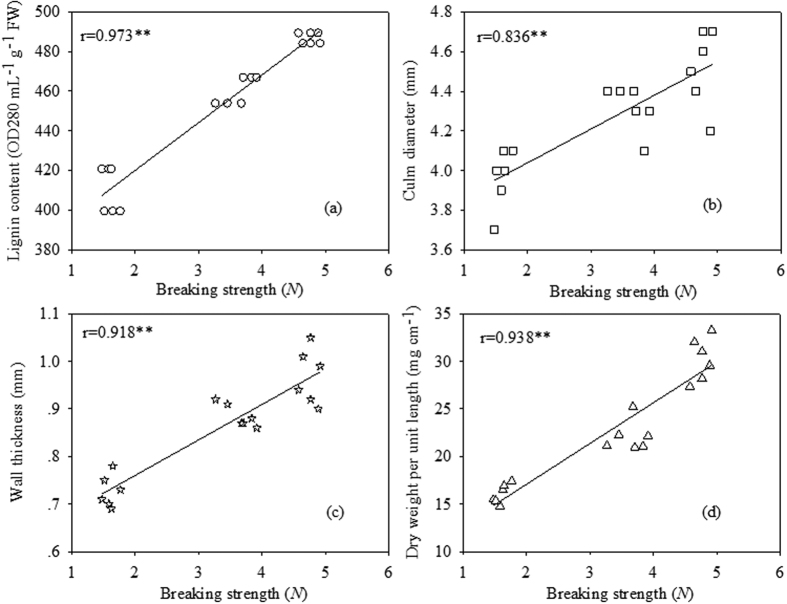
The relationship between the breaking strength and morphological characteristics of the second basal internode and the lignin content. (**a**), lignin content; (**b**), culm diameter; (**c**), wall thickness; (**d**), dry weight per unit length.

There was a significantly positive correlation between the breaking strength and lignin content of the second basal internode at the hard dough stage (Fig. 6a). Correlation analysis also demonstrated that the breaking strength of the second basal internode had a strong positive correlation with various morphological characteristics, including culm diameter, wall thickness and dry weight per unit length (Fig. 6b,c,d).

## Discussion

Lodging control in wheat is mainly determined by genetic factors, but it can also be modulated by plant density^34^,^40^. Increasing plant density is an effective cultivation practice to improve wheat grain yield; however, the plants will also be prone to lodging. A reasonable plant density is an important feature associated with stronger culm compared with the culm of overly large populations. Consistent with previous studies, we found that the breaking strength of the wheat second basal internode strongly increased with decreasing plant density^34^,^40^, thus decreasing the risk of lodging.

Plant density affects several processes during wheat culm development, such as morphological characteristics, anatomical structure, and the lignin biosynthesis inherent to these structures. In this study, we combined physiology, biochemistry, anatomy and molecular biology to clarify the mechanism through which plant density regulates the breaking strength of wheat culms, focusing on lignin biosynthesis. Our results allowed us to conclude that there is a relationship between plant density and the breaking strength of wheat culms. To understand the mechanism through which plant mechanical strength is controlled in wheat, we performed an in-depth analysis of different plant densities.

Culm morphological traits are related to lodging resistance^41^. Previous studies have suggested that stem diameter is a key factor in lodging resistance and that increasing the culm diameter in rice breeding programmes would thus improve lodging resistance in this crop^32^. In our study, we found that plant density had a significant effect on the culm diameter of the wheat second basal internode. With decreasing plant densities, the culm diameter of both cultivars increased in both growing seasons and exhibited a significantly positive (r = 0.836**) correlation with culm breaking strength. Previous reports have also agreed that culm wall thickness also has an important role in improving lodging resistance^12^. Significant correlations have been detected between resistance to lodging and wall thickness^42^. In this study, we concluded that decreasing the plant density increased the culm wall thickness. In addition, the dry weight per unit length, which represents the degree of culm filling, had a positive relationship with the culm breaking strength. This finding is similar to results obtained with rice culm, in which the degree of filling also significantly decreased with increasing population size^41^. Morphological features that provided the best indication of improved lodging resistance included increased culm diameter, wall thickness, and dry weight per unit length. These results support the hypothesis that larger populations have lower culm quality and a consequently increased risk of lodging in the case of wheat.

Anatomical stem characteristics affect lodging in wheat. Significant correlations were detected between lodging resistance and several anatomical features, including the width of the mechanical tissue layers and the width of stem walls^42^. A reduction in the mechanical strength of culms and negatively affected morphological characteristics in larger populations may reflect alterations in the cell wall structure. Therefore, we examined cell wall morphology using scanning electron microscopy to trace such alterations. In the D3 treatment, the number of mechanical tissues layers, especially those around the peripheral vascular tissues and under the epidermal tissue in culms, were reduced compared with D1 and D2 treatments, thus leading to weaker mechanical support in D3 plants. Scanning electron microscopy observations also revealed sclerenchyma and vascular bundle cells with thin walls in plants that received the D3 treatment. This finding is in striking contrast with that observed in the D1 treatment: the plants had thick-walled sclerenchyma and vascular bundle cells, in which the secondary wall occupied a large proportion of the cell lumen. The physiological factors associated with cell structure are unknown. Further studies are needed to investigate the physiological and genetic aspects of morphogenesis that are associated with the formation of secondary cell walls. Furthermore, staining with safranin O-fast green showed that the degree of lignification in D3 treatment was lower than that in D1 treatment. A high degree of lignification may be responsible for the stiff culm of wheat. Cortical tissues, especially sclerenchyma, are very important for enabling the culm to cope with bending stresses^42^. Our results suggest that the lower level of mechanical strength recorded for the D3 plants compared with the plants in the other treatments is likely to result from a defect in the cell wall thickening and lignification of the mechanical tissues, such as sclerenchyma, and vascular bundle elements.

A deficiency in the lignin biosynthesis in the secondary cell walls often leads to globally altered plant morphology, cell structure and even sterility^43^,^44^,^45^. In this study, the lignin content showed a significantly positive (r = 0.973**) correlation with the culm breaking strength. Lignin continuously increased between 0 and 35 days after the formation of the second internode in both cultivars, after which considerably reduced accumulation was observed during the later stages of stem development. This pattern of lignification is consistent with previous reports^12^,^46^. A similar developmental pattern of lignin deposition was observed in maize, in which a rapid increase in lignin content was observed along the progressive elongation stage^47^,^48^. Such a lignin accumulation pattern is consistent with the fact that lignification begins before the cessation of the elongation process. To determine whether the cellular phenotype, the degree of lignification and the reduction in mechanical strength in D3 plants resulted from altered lignin biosynthesis, we compared the lignin content among plant density treatments given that lignin is related to stem stiffness. Compared with the D1 and D2 treatments, the lignin content in D3 was significantly reduced. This result suggests that overly dense populations did not exhibit lignin accumulation.

Lignin accumulation and its composition (i.e., H-, G- and S-type monomers) are important factors influencing the breaking strength of wheat culm. It has been suggested that syringyl biosynthesis provides significant mechanical advantages to angiosperm species. However, the guaiacyl function in these plants involves conduction, not mechanical support^45^. Our data showed that guaiacyl units were the most abundant monomer in both cultivars. Furthermore, there was a greater abundance of syringyl units was compared with hydroxyphenyl units. Plant density had a strong effect on lignin monomer content and the proportion of each monomer. In this study, we showed that lignin composition also varies with plant density. With increased plant density, the content and proportion of H-type monomers increased at the expense of S-type monomers. It is likely that plants compete for energy in overly large populations; therefore, plants would choose a relatively low-energy monomer pathway, i.e., the H-type. We conclude that the S-type monomer plays an important role in the improved breaking strength of the wheat culm. To the best of our knowledge, this study is the first to determine lignin monomer variation with plant density and to elucidate the relationship between such variation and stem mechanical support.

To further clarify the regulation mechanism of lignin and lignin monomer accumulation according to plant density, transcriptomic data were used to determine several genes that are most likely to be involved in lignin biosynthesis in wheat stems at different developmental stages. Previous studies have reported that PAL, 4CL, CCR, CAD and COMT play key roles in lignin biosynthesis in wheat^49^,^50^,^51^,^52^,^53^. Regarding the lignin biosynthetic pathway genes, different transcript abundances were observed at different developmental stages. The relative expressions of *Ta*PAL, *Ta*4CL, *Ta*CCR, *Ta*CAD and *Ta*COMT genes were maintained at relatively high levels at approximately the same stage of fast lignin accumulation. Interestingly, the majority of the maximum expression occurred in young internodes followed by a decrease during the later stages of development, whereas lignin content increased at the latest maturity stages. A possible explanation is that a higher level of gene expression occurs in the early stages of stem development and that, even with subsequent down-regulation, lignification is maintained due to the stability of the lignin biosynthesis enzymes^54^. The S branch-specific gene (i.e., COMT) was down-regulated in the D3 treatment, which is consistent with the decreased S-type monomer content compared with the D1 treatment.

Based on our results, we suggest that a drastic reduction in lignin deposition caused by overly large populations normally impacts plant growth and development. The compression strength and pull strength of wheat stems would be about to change under different plant densities. The mechanical stress would increase under lower plant density. On one hand, this can present as increased the precursor products forming the lignin which provide the stiffness for plant body. On the other hand, due to the responses of plant to stress (e.g. wind stress), plant body would be induced to form defensive substances (lignin). This type of regulatory function in cultivation actually means that induced effect in morphology. A reasonable plant density significantly decreased the risk of lodging occurring, not only by altering the basal stem morphological traits but also by modifying lignin metabolism, especially the mRNA relative expression of genes in the basal internode. It also contributed to lignin accumulation and increasing stem stiffness. It should be noted that this study has examined only a few genes, and other middle pathway enzymes and genes (Supplementary Fig. S1) involved in lignin biosynthesis should be further analysed in subsequent studies.

In conclusion, we have described here, to the best of our knowledge, the first systematic study of the mechanisms regulated by plant density, especially regarding the effect of plant density on lignin biosynthesis and its relation to lodging resistance in wheat. The above mentioned results are valuable for enhancing lodging resistance in wheat. Therefore, we believe that this work can be considered as a reference study for future wheat cultivation.

## Methods

### Plant material and experimental design

Experiments were performed during two growing seasons from October 2012 to June 2013 and from October 2013 to June 2014 at the Shandong Agricultural University Farm, Tai’an, Shandong Province, China (36°09′ N, 117°09′E). The average content of the organic matter in the tillage layer was 18.11 g kg^−1^, and the total nitrogen determined by kjeldahl mentod (N). the rapidly available phosphorous determined by colorimetry method (P) and the rapidly available potassium contents determined by flame photometry (K) were 1.23 g kg^−1^, 17 mg kg^−1^ and 113 mg kg^−1^, respectively^55^. Initially, 120 kg of N ha^−1^, 100 kg of P_2_O_5_ ha^−1^ and 120 kg of K_2_O ha^−1^ were applied as a basal fertiliser before planting. In addition, 120 kg of N ha^−1^ was applied at the jointing stage (DC 30)^56^. Pests, diseases and weeds were controlled by appropriate chemical applications during the growing period. We chose two cultivars, shannong23 (SN23) and weimai8 (WM8), which are extensively cultivated. Three plant density levels (75, 225 and 375 plants m^−2^) corresponding to low (D1), medium (D2) and high (D3) density levels were established for the field experiment. The experimental plots were arranged in a factorial scheme, in a completely randomised block design with three replications in each treatment. The plot size was 3 × 3 m with 10 rows (0.25-m line spacing).

### Sampling and measurements

Sampling was performed after the formation of the second basal internode (DC 32). Representative samples of the second basal internode were collected every 7 days from plants growing in the middle of each plot; there were a total of eight collections. The stem sheath was removed from the samples before the samples were plunged directly into liquid nitrogen for at least 30 min, and then stored at −80 °C until analysis.

### Morphological characteristics of the second basal internode

Fifteen representative main stems were chosen for the measurement of the culm diameter, wall thickness, and dry weight of the second basal internode, according to the methods described by Wei *et al*.^57^. The diameter and wall thickness of the second basal internodes were measured at the internode mid region using a digital calliper with an accuracy of 0.001 mm.

### Breaking strength measurement

Breaking strength was measured at anthesis (DC 62), and at the milk (DC 72) and hard dough stages (DC 82) following the methods of Chen *et al*.^58^ and Peng *et al*.^12^ with slightly modifications. Measurements were obtained using a plant lodging tester (Hangzhou TOP Instrument, China). For these measurements, second basal internodes with the stem sheath removed and were placed on the supporting pillars at a distance of 5 cm. The tester was set perpendicular to the middle of the stem, which lodged gradually, and the breaking strength was measured when the culm internode was pushed to its breaking point. Breaking strength was expressed in Newtons (*N*).

### Histological analysis

Ten 10 days after anthesis, the middle section of the second basal internodes (approximately 0.5 cm in width) from each treatment were fixed in FAA (5 mL of 38% formaldehyde, 5 mL of glacial acetic acid and 90 mL of 70% ethanol) for 24 h, dehydrated with ethanol, and embedded in paraffin. Cross-sections of approximately 4-μm thick were obtained using a microtome (Leica, Germany). To visualise the degree of lignification among the treatments, sections were stained with 1% safranin-O for 1.5 h, washed with distilled water, discoloured in an ethyl series and then counterstained with 0.5% fast green for 1 min. Stained cross-sections were mounted on microscope slides and visualised using a Nikon DS-V3 microscope (Nikon, Japan). Lignified zones were stained red.

The middle sections of the second basal internodes were also fixed in a mixture of 2% paraformaldehyde and 2.5% glutaraldehyde in a 0.1% mol L^−1^ phosphoric acid buffer for 24 h at 4 °C and post-fixed in 1% OsO_4_ in a 0.1 mol L^−1^ phosphoric acid buffer for 30 min. After critical point drying, cross-sectioned samples were sputter-coated with gold and viewed using a scanning electron microscope (JEOL, Japan).

### Lignin determination

To assess lignin content, 0.3 g of fresh stem samples free of leaf sheaths were ground in liquid N_2_, washed five times with 80% ethanol to remove soluble metabolites, washed with acetone and dried in a drying oven. Acetyl bromide analysis was conducted following Peng *et al*.^12^. Samples were exposed to a 4:1 (v/v) mixture of acetic acid:acetyl bromide and incubated at 70 °C for 2 h. Then, the samples were cooled to room temperature and transferred to 50-mL volumetric flasks containing 2 M NaOH, acetic acid and 7.5 M hydroxylamine hydrochloride, which were added to terminate the reaction. The constant volume of each sample was completed with acetic acid. Sample absorbance was read at 280 nm by a spectrophotometer (Shimadzu UV-2450, Japan). Lignin content was expressed as OD_280_ mL^−1^ g^−1^ fresh weight (FW).

### Determination of lignin monomers

The hard dough developmental stage was chosen for the analysis of lignin monomer yields (H, G and S units), which were determined by alkaline nitrobenzene oxidation. The conventional nitrobenzene oxidation method was used with some modifications^59^. A total of 20 mg of protein-free cell wall was suspended in 2.5 mL of 2 M NaOH and 0.7 mL of nitrobenzene in a 5-mL vial with an aluminium cap. After mixing, the samples were heated in an autoclave at 127 °C for 2 h. The autoclave reactor was subsequently cooled to 70 °C, and the vials were taken out of the reactor and cooled by placing under running water. The reaction mixture was then centrifuged at 5,000 *g* for 2 min. The water phase on the top was transferred to a 10-mL centrifuge tube and then extracted with ethyl acetate (2 mL × 3). The organic phase was combined and dried with anhydrous Na_2_SO_4_ and the solvent was removed using a Termovap Sample Concentrator. The sample obtained was resuspended with 1.6 mL of the mobile phase (initial conditions) and then subjected to analysis with an Acquity ultra-performance liquid chromatography-electrospray tandem mass spectrometry system (UPLC-MS/MS) (Waters, Milford, MA, USA). Hydroxybenzaldehyde (H), vanillin (G) and syringaldehyde (S) were used as standards to quantify the monomers in the samples. Chromatographic separation was performed on a Waters Acquity BEH C18 column (1.7 μm, 2.1 × 100 mm) with a gradient elution (Supplementary Table S1). The mass spectra method is presented in Supplementary Table S2. The chromatogram graph is presented in Supplementary Fig. S2.

### Gene expression analysis

The gene expression encompasses three independent biological replicates of each treatment. For each biological replicate, three technical replicates of each PCR reaction were performed. Briefly, total RNA from the second basal internode was isolated using a modified Trizol extraction method and treated with DNase I to remove any contaminant genomic DNA. First-strand cDNA was synthesised from 1 μg of total RNA using the Prime Script RT Reagent Kit (TIANGEN, China). Quantitative real-time PCR was performed using the SYBR Green Master Mix kit in a Step One Plus instrument (Applied Biosystems, Singapore). The primers used in the present study are presented in Supplementary Table S3. We used the β-actin gene as an endogenous control to normalise the amount of template. The qRT-PCR protocol and the three-step thermal cycling protocol were performed following the manufacturers’ instructions as follows: pre-denaturation at 95 °C for 15 s followed by 40 cycles of denaturation at 95 °C for 10 s, an annealing temperature of 58 °C for 20 s and an extension temperature of 72 °C for 30 s. The baseline and threshold were adjusted according to the manufacturer’s recommendations. Melting curves were used to verify the specificity of amplification. The relative expression levels were calculated with the equation 2^−∆∆Ct^.

### Statistical analysis

Statistical analysis was performed using Data Processing System software 7.05 (DPS). The means and significant differences between treatments were separated using the least significant differences (LSD) test at 5% probability.

## Additional Information

**How to cite this article**: Zheng, M. *et al*. Manipulation of lignin metabolism by plant densities and its relationship with lodging resistance in wheat. *Sci. Rep.* **7**, 41805; doi: 10.1038/srep41805 (2017).

**Publisher's note:** Springer Nature remains neutral with regard to jurisdictional claims in published maps and institutional affiliations.

## Supplementary Material

Supplementary Information

## Figures and Tables

**Table 1:** Effects of plant densities on the breaking strength of the basal second internode of the two genotypes in the two growing seasons.

		2012–2013			2013–2014		
Cultivar	Treatment	A(*N*) (DC, 65)	M (*N*) (DC, 75)	H (*N*) (DC, 87)	A (*N*) (DC, 65)	M (*N*) (DC, 75)	H (*N*) (DC, 87)
	D1	7.92 ± 0.77b	6.52 ± 0.60b	4.77 ± 0.52b	7.38 ± 0.21b	6.32 ± 0.25b	4.65 ± 0.22b
SN23	D2	6.32 ± 0.72c	5.16 ± 0.34c	3.84 ± 0.89c	5.84 ± 0.31c	4.51 ± 0.18c	3.46 ± 0.38c
	D3	4.06 ± 0.43e	3.42 ± 0.53e	1.48 ± 0.68d	4.81 ± 0.54d	2.09 ± 0.34e	1.65 ± 0.53d
	D1	9.62 ± 1.05a	8.01 ± 0.69a	5.72 ± 0.57a	9.97 ± 0.39a	9.05 ± 0.06a	5.21 ± 0.54a
WM8	D2	7.56 ± 0.61b	6.62 ± 0.78b	4.17 ± 0.73bc	7.82 ± 0.49b	6.07 ± 0.32b	3.20 ± 0.13c
	D3	5.42 ± 0.28d	4.21 ± 0.54d	2.29 ± 0.86d	5.70 ± 0.49c	3.75 ± 0.25d	1.89 ± 0.21d
	Cultivars (C)	0.0095	0.0314	0.066	0.0709	0.0999	0.0997
P-value	Densities (D)	0.0036	0.0123	0.0092	0.0047	0.0077	0.0089
	C × D	0.5954	0.3262	0.5501	0.0173	0.0001	0.0001

Each value represents the means ± SE (n = 15) for the second basal internode of the representative wheat culm. Least significant difference testing (LSD) was performed to compare the means of the three experimental treatments, means followed by the same letters within each column are not significantly different at P < 0.05. *N*, Newton, A, Anthesis stage; M, Milk stage; H, Hard dough stage; SN23, shannong23; WM8, weimai8; DC, digital code^56^.

**Table 2:** Effects of plant densities on the characteristics of the second basal internode at the hard dough stage (DC, 87).

		2012–2013			2013–2014		
Cultivar	Treatment	D (mm)	WT (mm)	DW (mg cm^−1^)	D (mm)	WT (mm)	DW (mg cm^−1^)
	D1	4.48 ± 0.07b	0.92 ± 0.02b	28.19 ± 1.28b	4.65 ± 0.11b	1.01 ± 0.06ab	32.03 ± 1.46b
SN23	D2	4.21 ± 0.01bc	0.88 ± 0.02ab	21.05 ± 1.49c	4.37 ± 0.06c	0.91 ± 0.04bc	22.26 ± 1.63c
	D3	3.92 ± 0.12c	0.71 ± 0.03c	15.46 ± 1.18d	4.05 ± 0.03d	0.78 ± 0.04c	16.91 ± 0.97d
	D1	5.08 ± 0.12a	1.05 ± 0.05a	32.46 ± 1.97a	5.14 ± 0.05a	1.10 ± 0.12a	35.23 ± 2.75a
WM8	D2	4.89 ± 0.17a	0.89 ± 0.04b	22.69 ± 1.94c	4.78 ± 0.09b	0.97 ± 0.08ab	24.19 ± 2.16c
	D3	4.36 ± 0.09b	0.72 ± 0.06c	16.17 ± 2.08d	4.59 ± 0.08bc	0.88 ± 0.07bc	17.83 ± 1.43d
	Varieties (V)	0.014	0.1508	0.0125	0.0066	0.0128	0.0049
P	Densities (D)	0.0337	0.0001	0.0001	0.0141	0.0063	0.0001
	V × D	0.6066	0.2834	0.3158	0.7040	0.9555	0.7815

Each value represents the mean ± the SE (n = 15). The least significant difference test was performed to compare the means of the three treatments. Means followed by the same letters within each column are not significantly different at P < 0.05. D, diameter; WT, wall thickness; DW, dry weight; SN23, shannong23; WM8, weimai8.
