# Supplementary material for: Manipulation of lignin metabolism by plant densities and its relationship with lodging resistance in wheat
Source: Sci Rep. 2017 Feb 2;7:41805. doi: 10.1038/srep41805 (PMC5288770; doi:10.1038/srep41805)
Supplement: Supplementary Information [file srep41805-s1.pdf]

Supplementary material

Manipulation of lignin metabolism by plant densities and its relationship with lodging resistance in wheat

Mengjing Zheng[1*], Chen Jin[1*], Yuhua Shi[2*], Yanxia Li[1*], Dongqing Yang[1], Yongli Luo[1], Dangwei Pang[1], Xu Xu[1], Yanping Yin[1], Wenqian Li[1], Jun Ni[1], Yuanyuan Wang[1], Zhenlin Wang [1*] & Yong Li[1*]

[1]State Key Laboratory of Crop Biology, Ministry of Science and Technology, Shandong Agricultural University, Tai'an 271018, Shandong, P. R. China.

[2]Agricultural Bureau of Yanzhou District, Jining, Shandong, P. R. China.

*** Corresponding author.** E-mail addresses: zlwang@sdau.edu.cn; xmliyong@sdau.edu.cn.

Discussion

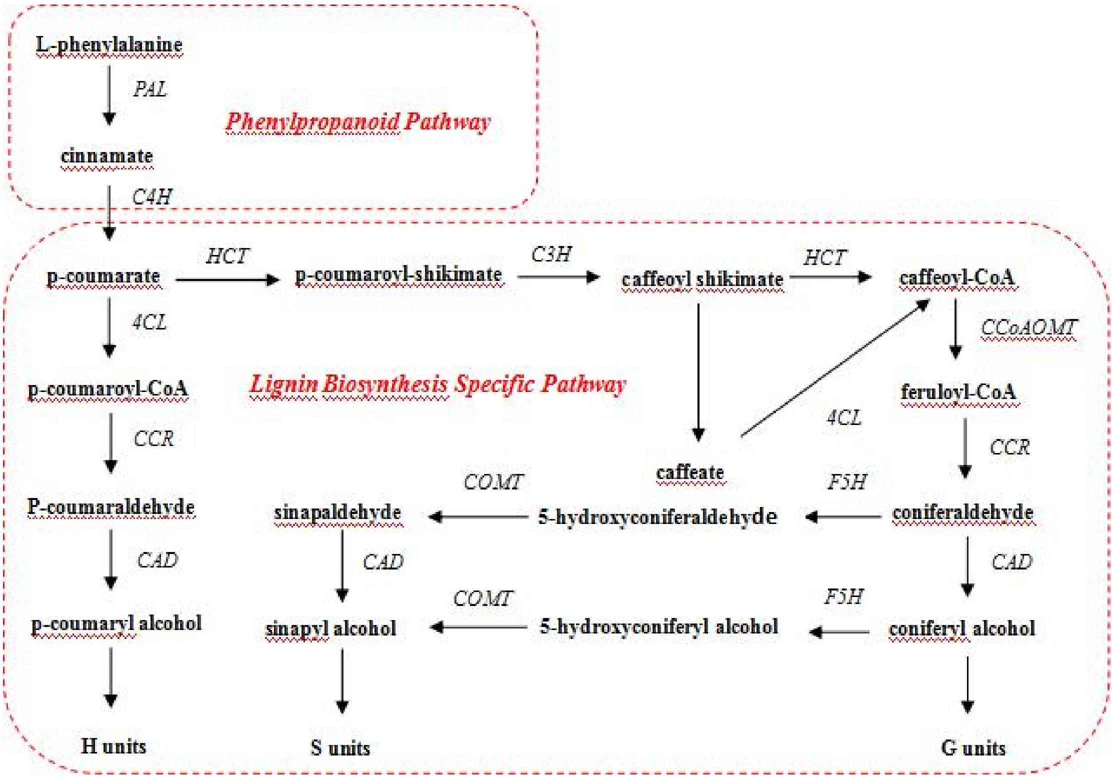

Fig. S1 Pathways for the biosynthesis of lignin monolignols. PAL, phenylalanine ammonia-lyase; C4H, cinnamate 4-hydroxylase; 4CL, 4-coumarate:CoA ligase; HCT, *p*-hydroxycinnamoyl-CoA shikimate/quinate hydroxycinnamoyl transferase; CCoAOMT, caffeoyl CoA *o*-methyl transferase; CCR, cinnamoyl CoA reductase; F5H, ferulic acid/coniferaldehyde/coniferyl alcohol 5-hydroxylase; COMT, caffeic acid3-*o*-methytransferase; CAD, cinnamyl alcohol dehydrogenase; H, *p*-hydroxyphenyl; G, guaiacyl; S, syringyl.

Methods

	Time (min)	Flow (mL/min)	%A (water)	%B (acetonitrile)	Total runing time (min)
1	Initial	0.500	95.0	5.0	Initial
2	0.50	0.500	95.0	5.0	6
3	3.00	0.500	75.0	25.0	6
4	3.50	0.500	10.0	90.0	6
5	4.00	0.500	10.0	90.0	6
6	4.10	0.500	95.0	5.0	6
7	6.00	0.500	95.0	5.0	6

Table S1 Chromatographic gradient conditions for the analysis of lignin monomers.

Compound name	Parent (m/z)	Daughter (m/z)	Dwell (s)	Cone (V)	Collision (V)
Hydroxybenzaldehyde	120.9681	92.1073	0.050	10	20
Vanllin	150.9681	92.0630	0.050	25	22
Vanllin	150.9681	136.0282	0.050	25	15
Syringaldehyde	180.9622	151.0684	0.050	10	20
Syringaldehyde	180.9622	166.0246	0.050	10	12

Table S2 Optimized mass spectra conditions.

Gene	Forward (5′→3′)	Reverse (5′ →3′)	GenBank No.
*Ta*PAL	*AGAGCACGGTGAAGAACACGGTA*	*ACACATACTCCCTGTCCAGAACTC*	JQ005112.1
Ta4CL	*GATCGAAGGCTCCGAAATCA*	*GACCTTGTGGATCCTCTTGTAG*	KF442977.1
*Ta*CAD	*GCAAGTCCTCCCAAATCCAC*	*CCTGTTTTCCTGAGGGTGTATCT*	GU563724.1
*Ta*CCR	*CCCAACAAGGTGCTCTGACG*	*CCTGTTTTCCTGAGGGTGTATCT*	DQ449508.1
*Ta*COMT	*TAACGAAGGGATGAAGAACC*	*GAAGTTGATGCCTTTTATGGT*	EF413031.1
β-actin	*CCTTCGTTTGGACCTTGCTG*	*AGCTGCTCCTAGCCGTTTCC*	AB181991.1

Table S3 Gene names and primer sequences used for Quantitative Real-time PCR.

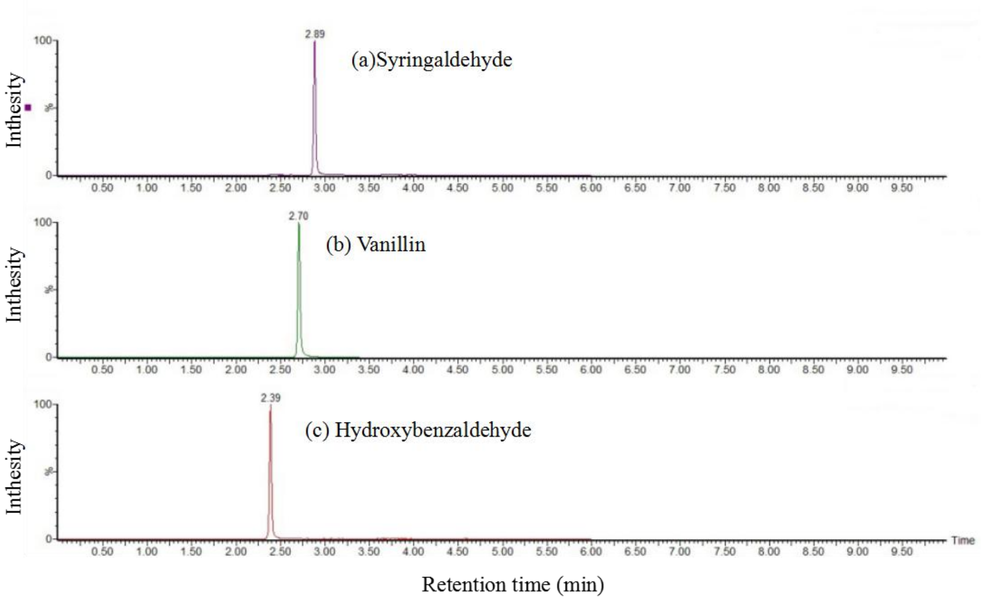

Fig. S2 UPLC-MS/MS analysis of lignin monomers by nitrobenzene oxidation. Total ion chromatograms of lignin monomers. (a), syringaldehyde; (b), vanillin; (c), hydroxybenzaldehyde.
